# A longitudinal study of changes in depressive symptoms and risk factors for congestive heart failure

**DOI:** 10.1192/bjo.2025.41

**Published:** 2025-05-09

**Authors:** Julia Gallucci, Justin Ng, Maria T. Secara, Brett D. M. Jones, Colin Hawco, M. Omair Husain, Nusrat Husain, Imran B. Chaudhry, Aristotle N. Voineskos, M. Ishrat Husain

**Affiliations:** Institute of Medical Science, University of Toronto, Toronto, Canada; Campbell Family Mental Health Research Institute, Centre for Addiction and Mental Health, Toronto, Canada; Department of Psychiatry, University of Toronto, Toronto, Canada; School of Health Sciences, Division of Psychology and Mental Health, University of Manchester, Manchester, UK; Mersey Care NHS Foundation Trust, Liverpool, UK; Department of Psychiatry, Ziauddin University, Karachi, Pakistan

**Keywords:** Congestive heart failure, depressive symptom changes, longitudinal study, low-to-middle-income countries, protective factors and risk factors

## Abstract

**Background:**

Depression is prevalent among patients with congestive heart failure (CHF) and is associated with increased mortality and healthcare use. However, most research on this association has focused on high-income countries, leaving a gap in knowledge regarding the relationship between depression and CHF in low-to-middle-income countries.

**Aims:**

To identify changes in depressive symptoms and potential risk factors for poor outcomes among CHF patients.

**Methods:**

Longitudinal data from 783 patients with CHF from public hospitals in Karachi, Pakistan, were analysed. Depressive symptom severity was assessed using the Beck Depression Inventory. Baseline and 6-month follow-up Beck Depression Inventory scores were clustered using Gaussian mixture modelling to identify separate depressive symptom subgroups and extract trajectory labels. Further, a random forest algorithm was used to determine baseline demographic, clinical and behavioural predictors for each trajectory.

**Results:**

Four separate patterns of depressive symptom changes were identified: ‘good prognosis’, ‘remitting course’, ‘clinical worsening’ and ‘persistent course’. Key factors related to these classifications included behavioural and functional factors such as quality of life and disability, as well as the clinical severity of CHF. Specifically, poorer quality of life and New York Heart Association (NYHA) class 3 symptoms were linked to persistent depressive symptoms, whereas patients with less disability and without NYHA class 3 symptoms were more likely to exhibit a good prognosis.

**Conclusions:**

By examining the progression of depressive symptoms, clinicians can better understand the factors influencing symptom development in patients with CHF and identify those who may require closer monitoring and appropriate follow-up care.

Depression is common among patients with congestive heart failure (CHF), with an estimated prevalence of 20–30%,^1,2^ making it one of the most frequent comorbid mental disorders in this population.^3^ Psychological health profoundly influences adherence to self-care practices,^4^ affecting both clinical outcomes and healthcare costs. Specifically, depression and depressive symptoms in patients with CHF contribute to increased healthcare expenditure,^3,5^ higher rates of hospital admission^6,7^ and heightened heart failure symptoms,^6^ as well as being risk factors for mortality.^3,7,8^ Depression and depressive symptoms in patients with CHF have also been linked to diminished quality of life,^3,9^ physical and social limitations,^6^ functional decline,^10^ worse New York Heart Association (NYHA) classification^10^ and decreased compliance to treatment regimens.^3^ However, few studies have investigated depression and depressive symptoms in patients with CHF, despite their high prevalence and substantial relationship with treatment adherence and outcomes.^3^

## Expanding research on depressive symptoms and CHF beyond high-income countries

Much of our current understanding of the biological, psychological and social factors underlying depression and depressive symptoms in CHF is derived from studies conducted in high-income countries, the findings of which may not extend to other regions of the world, given differential and complex sociodemographic factors.^11^ A recent review reported that social determinants including socioeconomic status, race and ethnicity influence depression rates in patients with CHF.^12^ There is also expected to be an increase in CHF burden in low-to-middle-income countries (LMICs), in which the aetiology of CHF has been shown to vary by income level,^13^ and factors such as access to healthcare, including cardiac rehabilitation services, may have a critical role in determining patient prognosis. In LMIC settings, these social determinants, combined with limited resources, may significantly affect the course and outcomes of depressive symptoms in CHF patients. The inclusion of diverse patient populations from LMICs could enable us to more comprehensively evaluate the relationship between depression and depressive symptoms in CHF and enhance the generalisability of our results.

## Exploring depressive symptom changes in CHF patients from LMICs

Previously, we prospectively investigated mortality, disability and health-related quality of life in depressed patients with CHF from Pakistan and found both a high rate of depression and a correlation between the severity of depressive symptoms and increased mortality.^14^ However, our earlier analyses used a binary classification of depression, which limited our ability to assess heterogeneous impairments among patients or account for potential differential changes in depressive symptoms. Other studies have identified depressive symptom trajectories in CHF patients from Western countries, including various patterns of persistence, improvement and worsening;^15–18^ however, to the best of our knowledge, there have been no reports of corresponding trajectories in CHF patients from LMICs. Investigating these changes in symptoms over time is crucial to enable personalised care and early intervention, especially in regions with limited healthcare resources. Understanding patterns of change could also lead to more targeted treatment, efficient resource allocation and culturally sensitive mental health strategies, thereby improving both psychological and cardiac outcomes.

The present investigation leveraged a large longitudinal data-set consisting of information about CHF patients from an urban setting in an LMIC (Karachi, Pakistan) to investigate changes in depressive symptoms over time. By applying machine learning techniques, we identified distinct depressive symptom patterns and explored associated clinical and demographic factors. Our findings offer novel insight into the dynamic nature of depressive symptoms in CHF patients, with a particular emphasis on low-resource settings, in which such research is scarce yet essential.

## Methods

### Data source

Data were obtained from a 6-month cohort study on CHF and depression, detailed in our prior publication,^14^ involving 1009 hospital patients recently diagnosed with CHF across Karachi, the most populous city in Pakistan, between 4 January 2010 and 31 December 31. Participants completed assessments at baseline and after 6 months, all of which had been validated for use in the Urdu language in Pakistan.^14^ Briefly, the following were assessed: social stress, using the Life Events Checklist, a 14-item tool assessing recent life events and stressors; perceived social support, using the Multidimensional Scale of Perceived Social Support, a questionnaire to evaluate levels of support from family, friends and significant others; quality of life, with the Euro Quality of Life Visual Analog Scale, which captures health status across five dimensions (mobility, self-care, usual activities, pain and/or discomfort, and anxiety and/or depression); disability, using the Brief Disability Questionnaire, a tool to evaluate functional limitations in daily activities; and depressive symptom severity, using the Beck Depression Inventory (BDI), a 21-item self-report questionnaire widely used to assess the severity of depressive symptoms. Health factors including the aetiology of CHF, presence of left ventricular failure, smoking status, ejection fraction and comorbidities (such as diabetes, chronic obstructive pulmonary disease, stroke, myocardial infarction, bypass surgery and renal disease) were also assessed. The NYHA classification was used to evaluate the severity of CHF, categorising participants into four classes (I–IV) on the basis of physical functional limitations (class I, no limitation; class II, slight limitation; class III, significant limitation; class IV, severe limitation, with symptoms even at rest).

### Ethics statement

The authors assert that all procedures contributing to this work comply with the ethical standards of the relevant national and institutional committees on human experimentation and with the Helsinki Declaration of 1975, as revised in 2013. All procedures involving human participants and/or patients were approved by the institutional review board of the Dow University of Health Sciences, Karachi (REB #038/10). Written informed consent was obtained from participants who met the inclusion criteria.

The authors affirm that this manuscript presents an honest, accurate, and transparent account of the study being reported. We confirm that no important aspects of the study have been omitted, and any discrepancies from the study as originally planned (and, where applicable, registered) have been fully explained.

### Statistical analysis

**Fig. 1:**
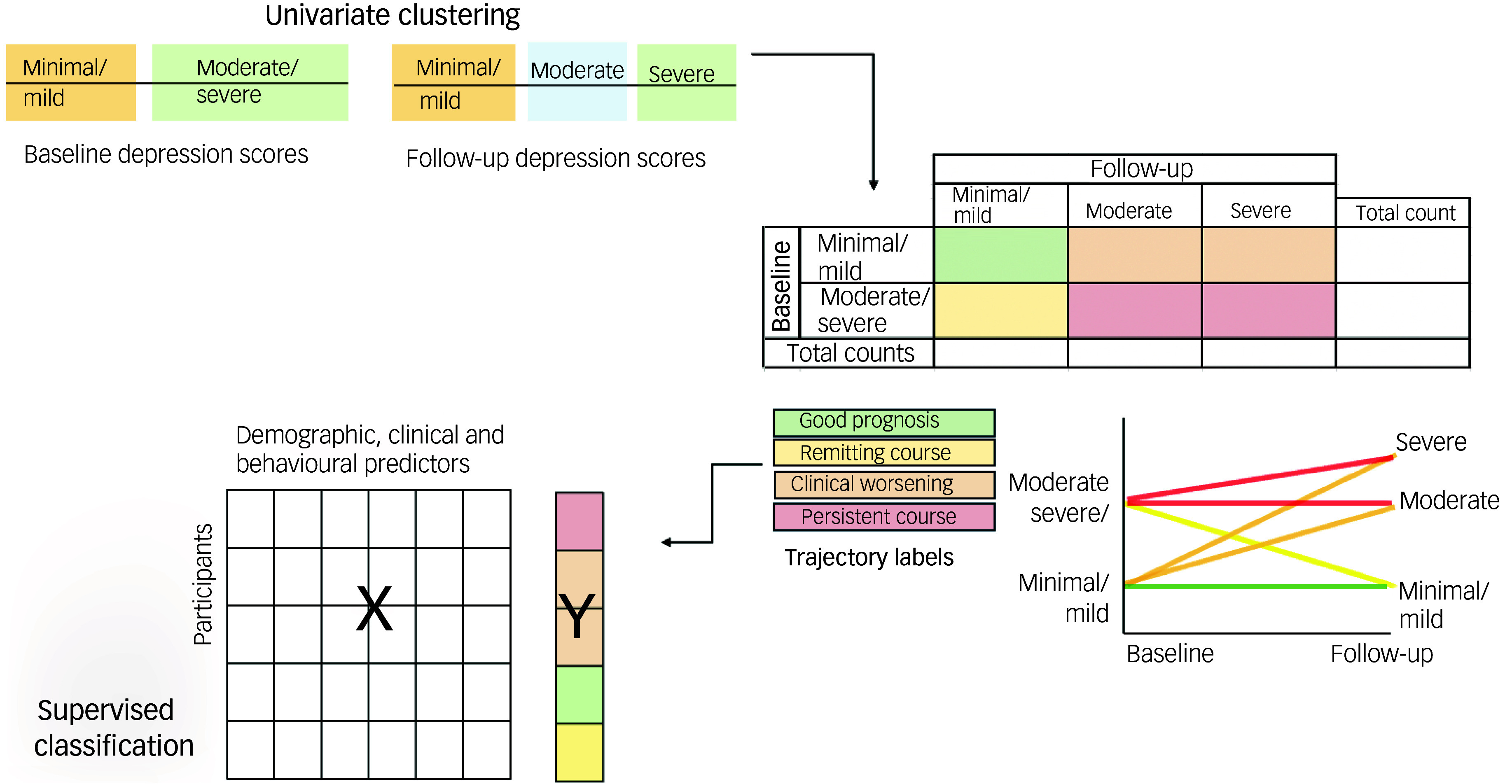
Schematic of analysis. Using univariate clustering, baseline and follow-up depressive symptom severity (Beck Depression Index) scores were analysed separately to identify clusters. Then, individual trajectory labels were assigned to participants on the basis of these clusters, and a supervised classifier was used to identify demographic, clinical and behavioural factors predictive of each trajectory label.

Our statistical methods workflow is outlined in Fig. 1. There were two main aims: (a) to identify changes in depressive symptoms; and (b) to identify factors contributing to these changes in depressive symptoms.

#### Aim 1: identification of patterns of depressive symptoms changes

To identify patterns of depressive symptom changes, a Gaussian mixture model (GMM) clustering approach was applied to baseline and follow-up depressive symptom severity scores (BDI scores) separately. Silhouette analysis was used to determine the optimal number of clusters. Silhouette scores measure the similarity of an object to its own cluster compared with others, with higher scores indicating better cluster separation. GMMs were fitted for cluster numbers ranging from 2 to 12, and the optimal number was selected on the basis of having the highest silhouette score. We also evaluated log-likelihood (a probabilistic measure of model fit) and penalised likelihood-based criteria (the Akaike information criterion and Bayesian information criterion) to assess model performance. These criteria provided additional perspectives on the trade-off between model fit and complexity, supporting a robust clustering solution. Labels were then extracted on the basis of the cluster assignment.

#### Aim 2: identification of factors contributing to changes in depressive symptoms

A random forest classifier was used to identify baseline features contributing to changes in depressive symptoms. A one-versus-rest approach was implemented, using separate binary classifiers to distinguish each class from the others. This method is particularly effective for imbalanced classes and provides insight into the specific characteristics of each group. All available baseline clinical and demographic variables were included in the model to ensure a data-driven approach to determining relevant factors. Baseline depressive symptom severity (BDI score) was omitted from our model to prevent data leakage, as clustering was based on this variable. Machine learning – specifically, the random forest method – was chosen because of its ability to handle complex, non-linear relationships between variables, which traditional methods such as regression may not capture. The random forest approach minimises uncertainty in prediction labels by using multiple decision trees, thereby reducing variance (i.e. sensitivity to idiosyncrasies in the training data). It also enables data-driven identification of important predictors; thus, it was a suitable choice given the variety of clinical and demographic factors we aimed to explore. This ensemble method allowed us to use bootstrapping and random feature selection to enhance the independence of individual trees and quantify feature importance, thereby providing clear indication of the key factors driving depressive symptom changes.

#### Hyperparameter optimisation

To improve model performance, we used a nested cross-validation approach with five inner folds; this enabled hyperparameter tuning by further splitting the training data into inner training and validation folds. We used the TPE (tree-structured Parzen estimator) algorithm to which randomly initialise sets of hyperparameters to evaluate, then adaptively selected subsequent sets on the basis of feedback to improve model performance. We conducted 500 evaluations, optimising hyperparameters for F1 score, the harmonic mean of precision and recall, where precision is the proportion of correctly predicted positive labels among all predicted positives, and recall is the proportion of correctly predicted positive labels among all actual positives. The number of trees was set to 500 to balance performance and computational cost. Key hyperparameters optimised included the splitting criterion (Gini impurity or Shannon information gain), class weight for imbalance, maximum tree depth, maximum features per split, and minimum samples per leaf and split.

#### Performance metrics

We used several metrics to comprehensively evaluate classifier performance, in addition to F1 score. Confusion matrices were normalised to show the percentage of true labels classified as each predicted label, and accuracy was calculated as the ratio of correctly identified labels to total number of labels. In addition, we assessed the area under the precision-recall curve and the area under the receiver operating characteristic curve to measure the trade-offs between precision and recall, and between true and false positive rates, respectively. Here, a true positive refers to correct identification of an individual who belongs to the target group (e.g. correct identification of a patient who has persistent depressive symptoms); precision measures how many of the predicted positives were actually correct, and recall measures how many of the actual positives were correctly identified. A higher area under the precision-recall curve indicates that the model is good at identifying true positives while minimising errors. The area under the receiver operating characteristic curve measures how well the model distinguishes between positive and negative cases, with a higher value indicating better overall performance. The outer loop of the nested cross-validation estimated model performance on unseen data, preventing overfitting during hyperparameter tuning and reducing the risk of biased performance estimates compared with a single random train–test split. Each model’s performance on new data was estimated using ten folds with five repetitions, which required multiple splits of the data into training and testing folds. By averaging performance metrics across folds, we could therefore obtain an unbiased assessment of the model’s overall effectiveness.

#### Feature importance and directionality

Feature importance was evaluated by quantifying each feature’s capacity to reduce impurity, thereby decreasing label uncertainty during decision-tree construction. The reduction in impurity attributed to each feature was averaged across all trees in the random forest to determine its overall importance.

Although feature importance quantifies the extent to which each feature contributes to a prediction, it does not convey the direction of this influence. Shapley additive explanations (SHAP) values were used to address this.^19^ SHAP values provide a detailed account of each feature’s contribution to a prediction. Each feature is assigned a SHAP value for each prediction, reflecting both the magnitude and direction of its impact. A higher SHAP value indicates a stronger influence of the feature on the prediction. A positive SHAP value means an increase in the feature’s value drives the prediction towards the current class, whereas a negative SHAP value means it moves the prediction away from the current class.

#### Statistical testing

To assess whether the models predicted changes in depressive symptoms better than statistical chance, we performed non-parametric permutation testing on the performance metric for each model. Specifically, we conducted statistical testing on F1 score to maintain consistency with hyperparameter optimisation. To determine whether each feature contributed more to the prediction than statistical chance, we performed non-parametric permutation testing on the feature importance for each model. This involved randomly shuffling class labels outside the cross-validation framework and repeating cross-validation with the same previously optimised hyperparameters. We repeated this 1000 times, generating permuted null distributions to compare the true F1 and feature importance scores. We used one-sided tests to determine the numbers of permuted F1 and feature importance scores that exceeded the true scores at a significance level of α = 0.05. To correct for multiple comparisons, we separately conducted Benjamini–Hochberg *p*-value false discovery rate (pFDR) correction^20^ across the performance metric tests (4 × 1 comparisons) and feature importance tests (4 × 32 comparisons).^21^

### Code sharing

All code used for statistical analyses and generating figures can be found at https://github.com/juliagallucci/CHF_Depressive_Symptoms.

## Results

### Participant characteristics

A total of 783 individuals diagnosed with CHF were included in the analysis; all of these individuals had complete data, including baseline and follow-up BDI scores, as well as all baseline demographic, behavioural and clinical information. Participant demographics and clinical scores are detailed in Table 1.

**Table 1:** Participant’s demographics and clinical score (*n* = 783)

Demographics	Mean	s.d. [range]
Age, years	57.25	7.15 [46–82]
Monthly income, PKR	14,700.26	10,806.73 [0–90,000]
LEC	8.45	3.88 [1–15]
MSPSS		
Significant other	13.02	8.40 [4–28]
Family	12.25	8.36 [4–28]
Friends	10.83	8.21 [4–28]
EQVAS	40.98	11.25 [15–78]
BDQ	9.10	3.20 [1–20]

	*n*	%
Sex		
Female	248	31.67
Male	535	68.33
Education level^a^		
None	279	35.63
Primary	295	37.68
Metric	154	19.67
Graduate	55	7.02
Marital status		
Married	710	90.68
Single	29	3.70
Separated	13	1.66
Widowed	31	3.96
Employment		
Unemployed	67	8.56
Employed	397	50.70
Retired	93	11.88
Homemaker	226	28.86
History of depression		
No	697	89.02
Yes	86	10.98
LVF		
No	70	8.94
Yes	713	91.06
Ischaemic cause of CHF		
No	41	5.24
Yes	742	94.76
Diabetes		
No	346	44.19
Yes	437	55.81
COPD		
No	753	96.17
Yes	30	3.83
Stroke		
No	754	96.30
Yes	29	3.70
Renal disease		
No	725	92.59
Yes	58	7.41
Smoking		
No	532	67.94
Yes	251	32.06
Prior bypass surgery		
No	726	92.72
Yes	57	7.28
Prior infarction		
No	149	19.03
Yes	634	80.97
Ejection fraction		
I	207	26.44
II	515	65.77
III	61	7.79
NYHA class		
II	378	48.28
III	368	47.00
IV	37	4.73

BDQ, Behavioral Development Questionnaire; CHF, congestive heart failure; COPD, chronic obstructive pulmonary disease; EQVAS, Euro Quality of Life Visual Analogue Scale; LEC, Life Events Checklist; LVF, left ventricular fraction; MSPSS, Multidimensional Scale of Perceived Social Support; NYHA, New York Heart Association Functional Classification.

a. Primary education includes grades 1–6, metric education refers to secondary schooling (grades 9–10) and graduate education involves higher studies (Bachelor’s degree or equivalent).

### Identification of changes in depressive symptoms

#### Clustering at baseline and follow-up

**Fig. 2:**
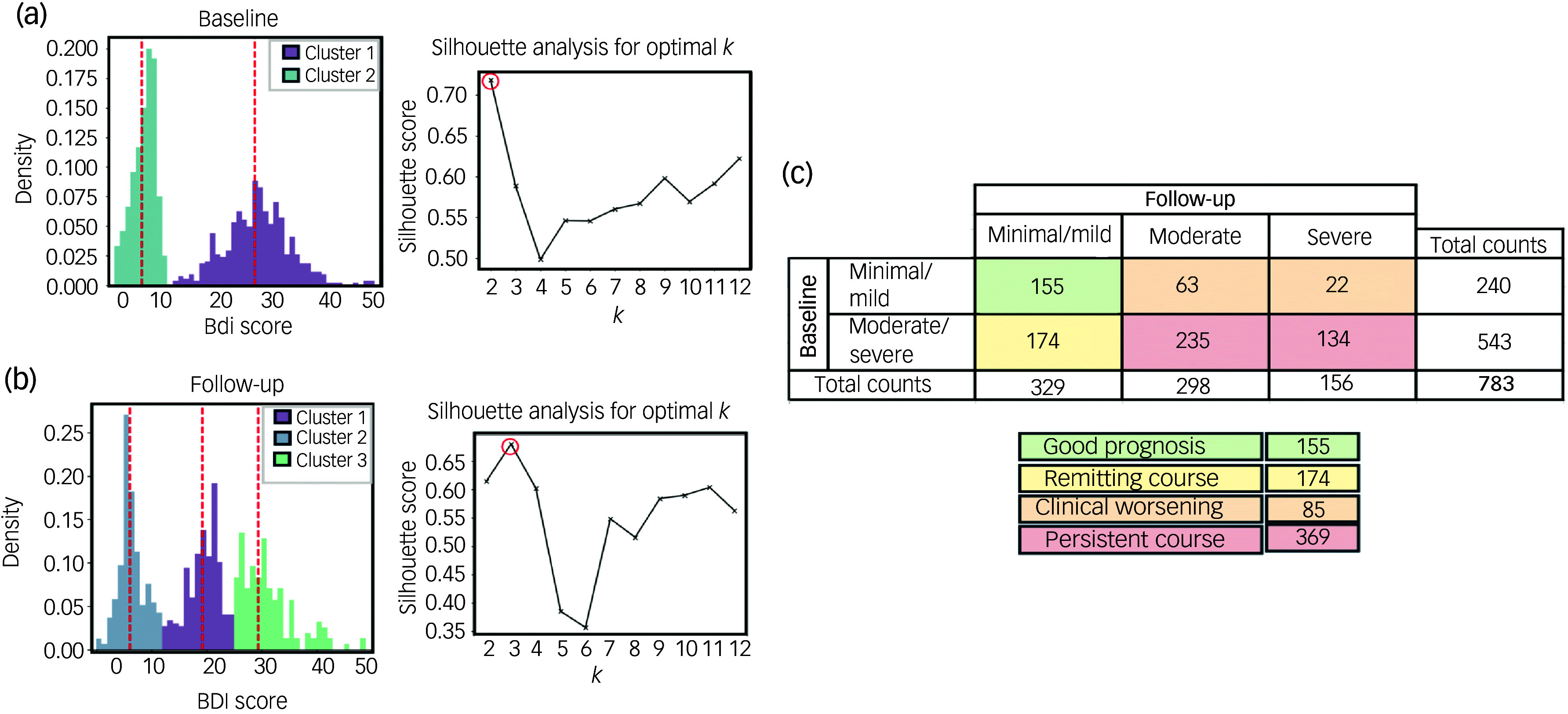
Depressive symptom changes in patients with congestive heart failure. Gaussian mixture model clustering and silhouette analysis were used to determine the optimal numbers of clusters (*k*) at (a) baseline and at (b) 6-month follow-up. (c) On the basis of these analyses, 6-month trajectory labels for depressive symptoms were derived. BDI, Beck Depression Index.

Using the GMM algorithm, we identified two separate participant clusters on the basis of depressive symptom severity (BDI score) at baseline (minimal/mild (30.65%) and moderate/severe (69.35%) depressive symptom severity) by taking the clustering with the highest silhouette score (0.72) (Fig. 2(a)). Log-likelihood, Akaike information criterion and Bayesian information criterion values were consistent across solutions with numbers of clusters ranging from two to nine, but the silhouette-score-based solution aligned best with our visual inspection of the score distribution (Supplementary Fig. 1 available at https://doi.org/10.1192/bjo.2025.41). At follow-up, three separate patient clusters emerged, consisting of patients with ‘minimal/mild’ (42.02%), ‘moderate’ (38.06%) and ‘severe’ (19.92%) depressive symptom severity; again, the clustering was based on the silhouette score (highest score: 0.68) (Fig. 2(b)). As at baseline, the log-likelihood, Akaike information criterion and Bayesian information criterion values remained stable across solutions with 2–7 clusters, and the silhouette score provided the most visually coherent clustering (Supplementary Fig. 1). There was a high degree of overlap in depressive symptom severity between the minimal/mild cluster at baseline and that at follow-up, indicating consistency in our interpretation of changes over time (Supplementary Fig. 2).

#### Applying changes to depressive symptom labels

Four patterns of depressive symptom changes were identified: ‘good prognosis’ (minimal/mild at both baseline and follow-up; *n* = 155), ‘remitting course’ (moderate/severe at baseline and minimal/mild at follow-up; *n* = 174), ‘clinical worsening’ (minimal/mild at baseline and either moderate or severe at follow-up; *n* = 85) and ‘persistent course’ (moderate/severe at both baseline and follow-up; *n* = 369) (Fig. 2(c)). Although two clusters were identified at baseline (minimal/mild and moderate/severe) and three clusters at follow-up (minimal/mild, moderate and severe), the moderate and severe clusters at follow-up were combined under a single label, as they both corresponded to levels of depressive symptomatology that generally necessitate clinical intervention. Demographic and clinical characteristics for each group are presented in Supplementary Table 1.

### Factors contributing to changes in depressive symptoms

**Fig. 3:**
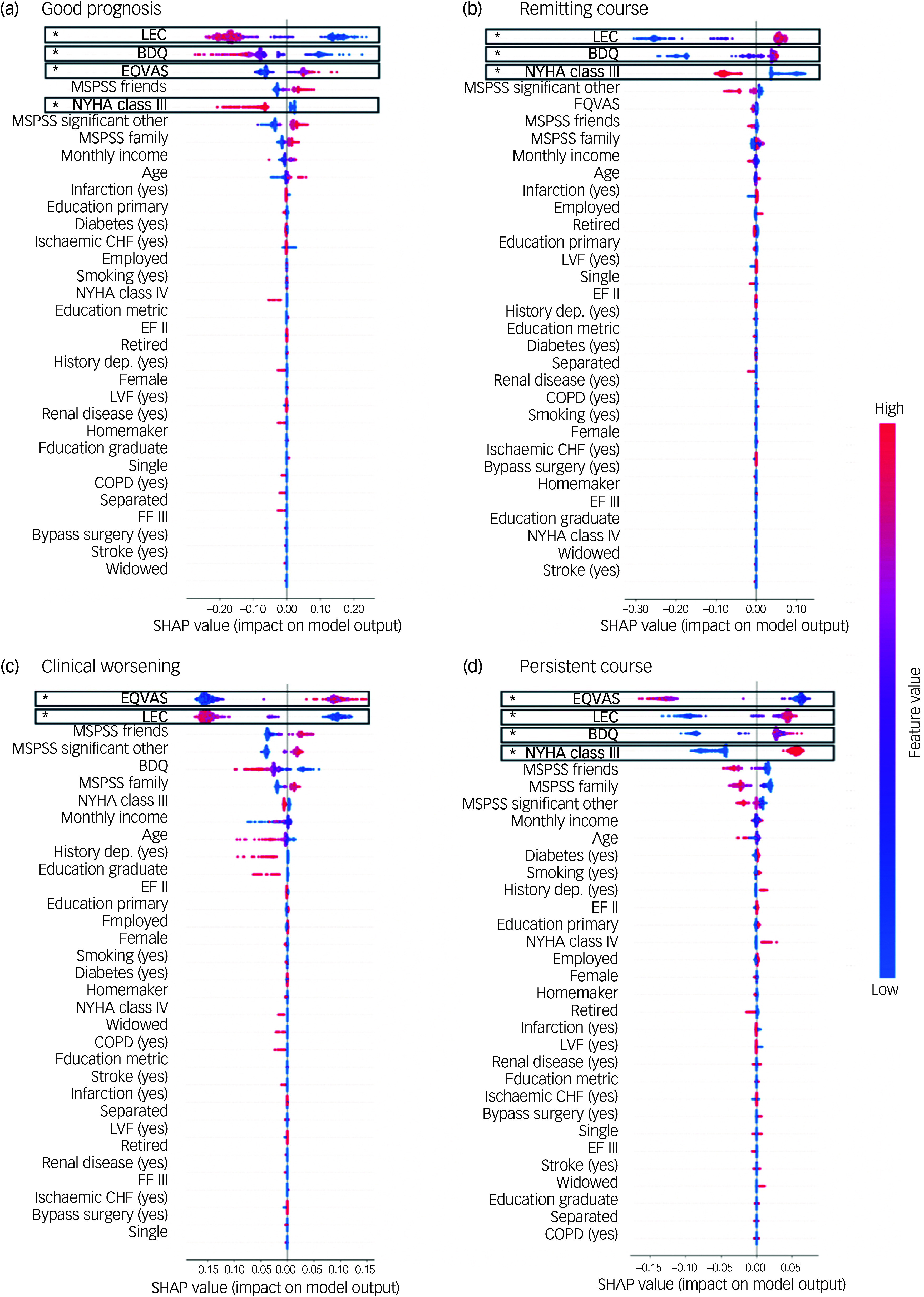
Shapley additive explanations (SHAP) values for one-versus-rest random forest models. SHAP values are shown for predictors of (a) good prognosis, (b) remitting course, (c) clinical worsening and (d) persistent course. Positive SHAP values indicate a positive contribution to the prediction, whereas negative values indicate a negative contribution. Predictors are ordered on the basis of impurity-based feature importance, with significance indicated (asterisk indicates positive FDR < 0.05). BDQ, Behavioral Development Questionnaire; CHF, congestive heart failure; COPD, chronic obstructive pulmonary disease; EQVAS, Euro Quality of Life Visual Analogue Scale; LEC, Life Events Checklist; LVF, left ventricular fraction; MSPSS, Multidimensional Scale of Perceived Social Support; NYHA, New York Heart Association Functional Classification; EF II, ejection fraction II; EF III, ejection fraction III.

The initial analysis involved implementing the random forest classifier with default hyperparameters. Although the model accurately predicted the good prognosis and persistent course patterns, it frequently misclassified clinical worsening and remitting course (Supplementary Fig. 3). To address this, we optimised hyperparameters to improve the F1 score, which was particularly sensitive to these misclassifications. Given the relatively good classifier performance (all *P* < 0.0099), with the confusion matrix showing that the majority of true labels were correctly classified and there were few misclassifications (Supplementary Fig. 4), we investigated the most critical features at baseline for each outcome using the F1-optimised models (Fig. 3).

Key features associated with each depressive symptom trajectory were as follows. Good prognosis had a positive association with quality of life (feature importance 0.15; pFDR = 0.014) and negative associations with social stress (feature importance 0.39; pFDR = 0.014), disability (feature importance 0.18; pFDR = 0.014) and NYHA class 3 (feature importance 0.06; pFDR = 0.049) (Fig. 3(a)). Remitting course had positive associations with social stress (feature importance 0.40; pFDR = 0.014) and disability (feature importance 0.28; pFDR = 0.014) and a negative association with NYHA class 3 (feature importance 0.13; pFDR = 0.026) (Fig. 3(b)). Clinical worsening had a positive association with quality of life (feature importance 0.32; pFDR = 0.014) and a negative association with social stress (feature importance 0.31; pFDR = 0.014) (Fig. 3(c)). Finally, persistent course had positive associations with social stress (feature importance 0.20; pFDR = 0.014), disability (feature importance 0.17; pFDR = 0.046) and NYHA class 3 (feature importance 0.12; pFDR = 0.049) and a negative association with quality of life (feature importance 0.26; pFDR = 0.014) (Fig. 3(d)).

**Fig. 4:**
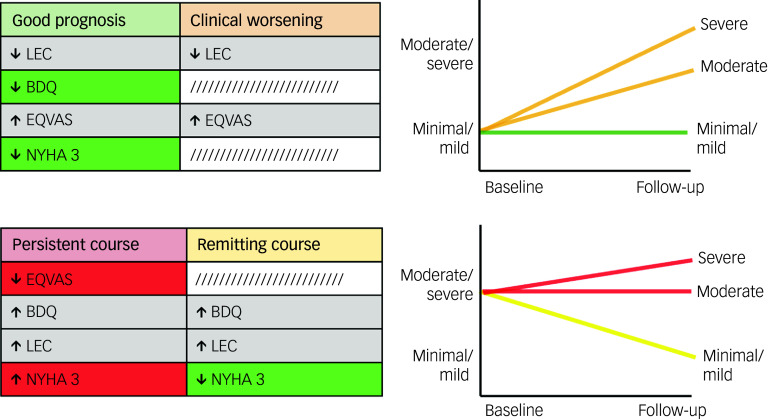
Significant predictors and their associations with changes in depressive symptoms, grouped based on shared baseline depressive symptom severity scores. Risk factors (red) are uniquely linked to poorer outcomes, protective factors (green) are uniquely linked to better outcomes, and common factors (grey) are associated with both. BDQ, Behavioral Development Questionnaire; EQVAS, Euro Quality of Life Visual Analogue Scale; LEC, Life Events Checklist; NYHA, New York Heart Association Functional Classification.

To better interpret these factors, we performed *post hoc* groupings of changes in depressive symptoms according to baseline depressive symptom severity scores (Fig. 4). Groups with minimal baseline depressive symptom severity (good prognosis and clinical worsening) were positively associated with quality of life and negatively associated with and social stress. Uniquely, maintaining minimal depressive symptom severity over time (good prognosis) was negatively associated with disability and NYHA class 3. By contrast, groups with moderate/severe baseline depressive symptom severity (persistent course and remitting course) had positive associations with both disability and social stress. Uniquely, poor prognosis over time (persistent course) was negatively associated with quality of life and positively associated with NYHA class 3, whereas symptom improvement over time (remitting course) was negatively associated with NYHA class 3.

## Discussion

In the present investigation, we leveraged a longitudinal data-set corresponding to CHF patients from an urban setting in an LMIC that had also been assessed for depressive symptoms. We aimed to uncover patterns of depressive symptom severity over time and explore related baseline demographics and clinical factors. We identified four separate patterns of depressive symptom changes: good prognosis, remitting course, clinical worsening and persistent course. Key contributors to these changes included quality of life, social stress, disability and CHF classification. Specifically, minimal baseline depressive symptom severity was linked to better quality of life and lower social stress, whereas lower disability levels and NYHA class other than 3 were associated with good longitudinal outcomes. Conversely, moderate/severe baseline depressive symptom severity was linked to higher disability and social stress, whereas lower quality of life and NYHA class 3 symptoms were associated with more persistent poor outcomes.

Our study identified four separate patterns of depressive symptom changes in this sample. In approximately half of the patients presenting with depressive symptoms at baseline, these persisted at follow-up, whereas around 40% either maintained minimal symptoms or exhibited clinical improvement. A small percentage showed worsening of depressive symptoms after 6 months. These results are consistent with findings of previous research on depressive symptom trajectories in Western countries,^15–18^ indicating that a significant portion of CHF patients experience persistent depressive symptoms, and, among those who do show changes, the majority tend to improve rather than decline, irrespective of setting.

Poorer longitudinal outcomes were linked to lower quality of life and higher CHF functional classification (NYHA class 3), indicating moderately severe cardiovascular disease with physical activity limitations. These findings are consistent with those of previous studies linking higher NYHA class to increased psychological distress, including anxiety,^22^ depression^23,24^ and overall reduced quality of life.^25,26^ Given the subjective nature of NYHA, which is based on patients’ experience of limitations in daily activities due to cardiac function,^22,26^ persistent depressive symptoms may distort illness perception, leading to an overemphasis on functional limitations. This interplay may worsen CHF patients’ perception of heart failure, leading those with persistent depressive symptoms to report greater limitations and poorer functioning, which in turn could perpetuate their depressive symptoms. It has previously been shown that the perception of symptom burden significantly affects mood-related symptoms (e.g. anxiety and depression) in patients with CHF.^27,28^

Lower levels of disability and non-severe CHF functional classification (i.e. NYHA class other than 3) emerged as protective factors against poorer longitudinal outcomes. Patients with changes in depressive symptoms characterised by worsening or persistent clinical symptoms lack these protective factors, which may lead to less favourable outcomes. Increased disability is associated with decreased quality of life and is an independent predictor of mortality.^29,30^ In addition, level of disability is directly correlated with NYHA functional classification and has been independently linked to cognitive dysfunction in CHF patients.^31^ These associations can impair patients’ ability to adhere to treatments, manage self-care and make informed health-related decisions,^32^ potentially contributing to a cycle of worsening physical and psychological health outcomes. Changes in depressive symptoms with more favourable outcomes (good prognosis and remitting course) were negatively associated with NYHA class 3 symptoms and disability, particularly in the good prognosis group. These findings suggest that level of disability, which affects physical and cognitive function as well as emotional well-being, may directly influence longitudinal outcomes and mediate the transition between favourable and unfavourable changes. Interventions such as resistance training and personalised exercise programmes could help to reduce physical disability^33,34^ and improve cognitive functioning.^35^ Exercise has also shown efficacy in reducing depression^36^ and thus may improve patient trajectories for individuals with CHF experiencing depressive symptoms.

This study had some limitations. Our analyses lacked an independent data-set for evaluation of the generalisability of the results. However, cross-validation and permutation procedures were used to mitigate the risk of unstable findings. In addition, this study was conducted as a secondary analysis and therefore was not originally designed prospectively to address the proposed aims. For instance, our data collection was limited to baseline and 6-month follow-up assessments. Serial assessments of depressive symptoms spanning this period would enable a more nuanced understanding of individuals’ true depressive symptom trajectories. The association between more severe NYHA class and depressive symptoms could indicate either that severe CHF causes depression in patients with significant functional impairment, or that depressive symptoms worsen heart failure symptoms and functional impairment. The present study could not establish the causality of this complex bidirectional relationship; further analysis is required to determine whether this association contributes to the link between depression and increased mortality in CHF patients.^37^ Last, although the BDI is a widely accepted measure of depressive symptom severity in adults,^38^ it lacks the diagnostic capabilities of gold-standard instruments such as the DSM or Mini International Neuropsychiatric Interview. This limitation, coupled with the absence of assessments of other psychopathologies such as anxiety or substance use disorders, restricted our ability to fully attribute observed outcomes to depression alone. Further, some BDI items overlap with physical health issues, including fatigue, poor sleep and somatic concerns; this could have increased depressive symptom scores in patients with severe CHF.^39^ Although psychological items on the BDI are less likely to be affected by such an overlap, this possibility should be considered when interpreting the findings. Inclusion of a broader range of assessments could have provided a more comprehensive understanding of how these factors interact with physical health outcomes.

The results of the present study underscore the heterogeneous nature of changes in depressive symptoms over time among CHF patients in LMICs, revealing a complex interplay of contributing risk and protective factors. These findings are strengthened by our having used a data-driven clustering approach, which led to a set of separate trajectory clusters, rather than a binary classification of depression status. This method provided a more nuanced understanding of changes in depressive symptoms by identifying natural groupings within the data, leading to more accurate and personalised insights into patient outcomes than might have been possible using a depressed versus non-depressed dichotomy. In addition, the use of a random forest classifier, a method robust to overfitting and capable of handling large imbalanced data-sets, enabled us to uncover meaningful predictors for each trajectory. Such risk factors must be carefully considered to enhance our understanding of which patients are at heightened risk of adverse outcomes, which in turn could facilitate early identification and initiation of appropriate interventions to maximise benefits. Furthermore, our findings may inform clinical trials of interventions to prevent depression and effectively manage diagnosed cases. For example, given the relationship between physical disability and depression, patients at high risk could be enrolled in more intensive rehabilitation and exercise programmes. This is particularly important given the significant impact of depression on mortality in CHF patients,^3,7,8^ as well as the additional suffering associated with comorbid conditions.^40^

## Supporting information

Gallucci et al. supplementary material Gallucci et al. supplementary material

## Data Availability

The data supporting the findings of this study are available upon request from the senior author, M.I.H. These data are not publicly available owing to the presence of information that could compromise the privacy of the research participants. All code used for statistical analyses and generating figures is freely accessible at https://github.com/juliagallucci/CHF_Depressive_Symptoms.

## Supplementary material

The supplementary material is available online at https://doi.org/10.1192/bjo.2025.41
