# Supplementary material for: A longitudinal study of changes in depressive symptoms and risk factors for congestive heart failure
Source: BJPsych Open. 2025 May 9;11(3):e93. doi: 10.1192/bjo.2025.41 (PMC12089807; doi:10.1192/bjo.2025.41)
Supplement: Gallucci et al. supplementary material [file S2056472425000419sup001.docx]

**Supplemental Material for Gallucci et al., A longitudinal study of changes in depressive symptoms and risk factors in congestive heart failure**

Supplemental Figures

Figure S1. AIC, BIC, and log-likelihood values across clustering solutions from 2

to 12 clusters at baseline and follow-up

Figure S2. Evaluation of depressive symptom severity in the minimal/mild cluster

across time points

Figure S3. Confusion Matrices and Performance Metrics of One-vs-Rest Binary

Classifiers with default hyperparameters.

Figure S4. Confusion Matrices and Performance Metrics of One-vs-Rest

Binary Classifiers Optimized for F1.

Supplemental Table

Table S1. Participant’s Demographics and Clinical Score Stratified by Trajectory

**Supplemental Figures**


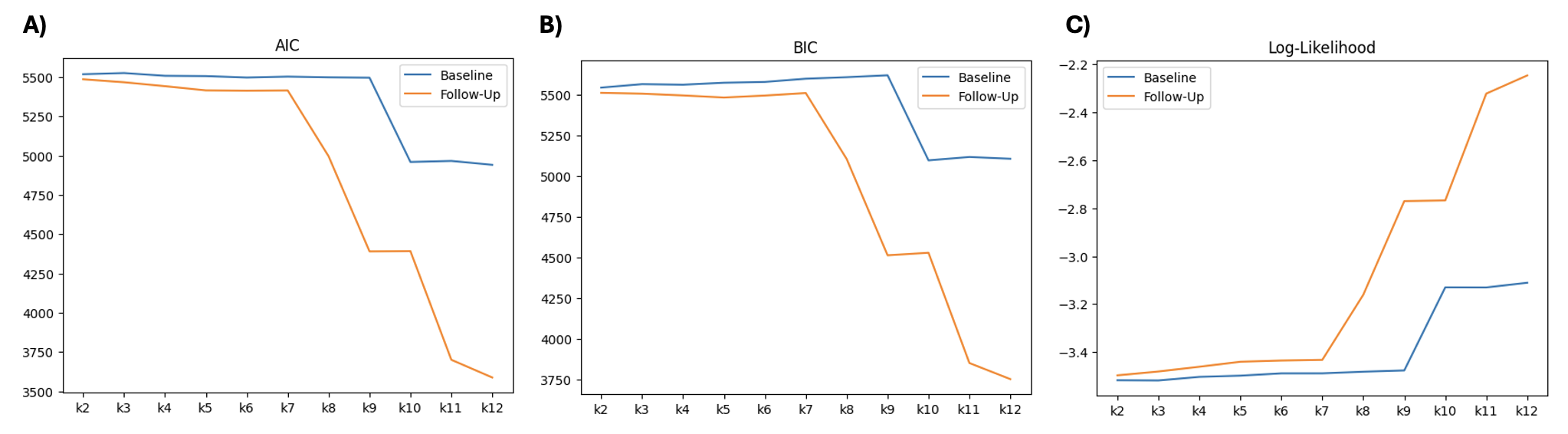


**Figure S1: AIC, BIC, and log-likelihood values across clustering solutions from 2 to 12 clusters at baseline and follow-up.**


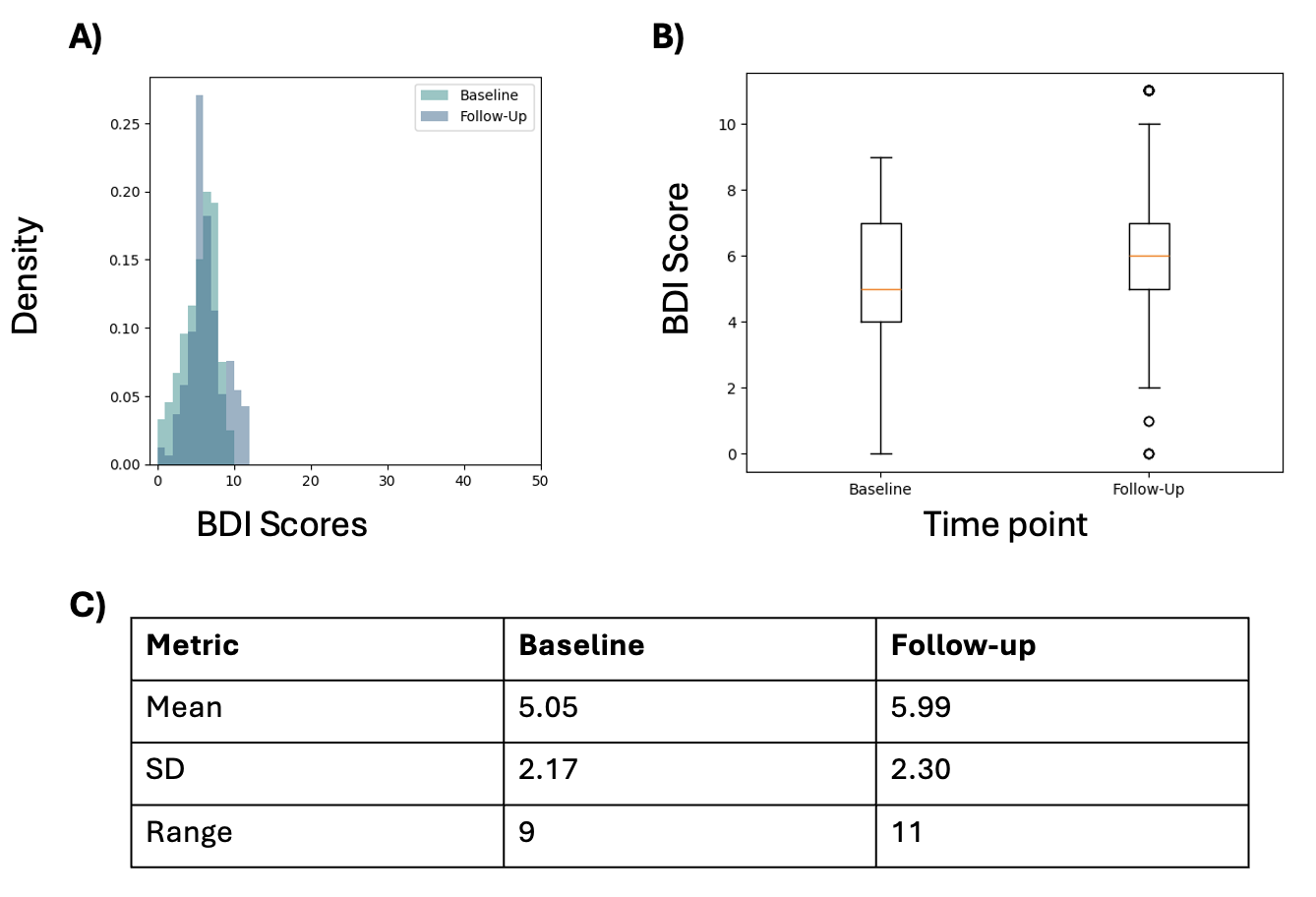


**Figure S2: Evaluation of depressive symptom severity in the minimal/mild cluster across time points.** A) Overlapping histograms of depressive symptom severity at baseline and follow-up for individuals in the minimal/mild cluster, illustrating the distribution and degree of overlap. B) Boxplot comparing the range and distribution of depressive symptom severity scores at both time points for the minimal/mild cluster.
 C) Summary table showing the mean, standard deviation, and range of depressive symptom severity scores for the minimal/mild cluster at baseline and follow-up.


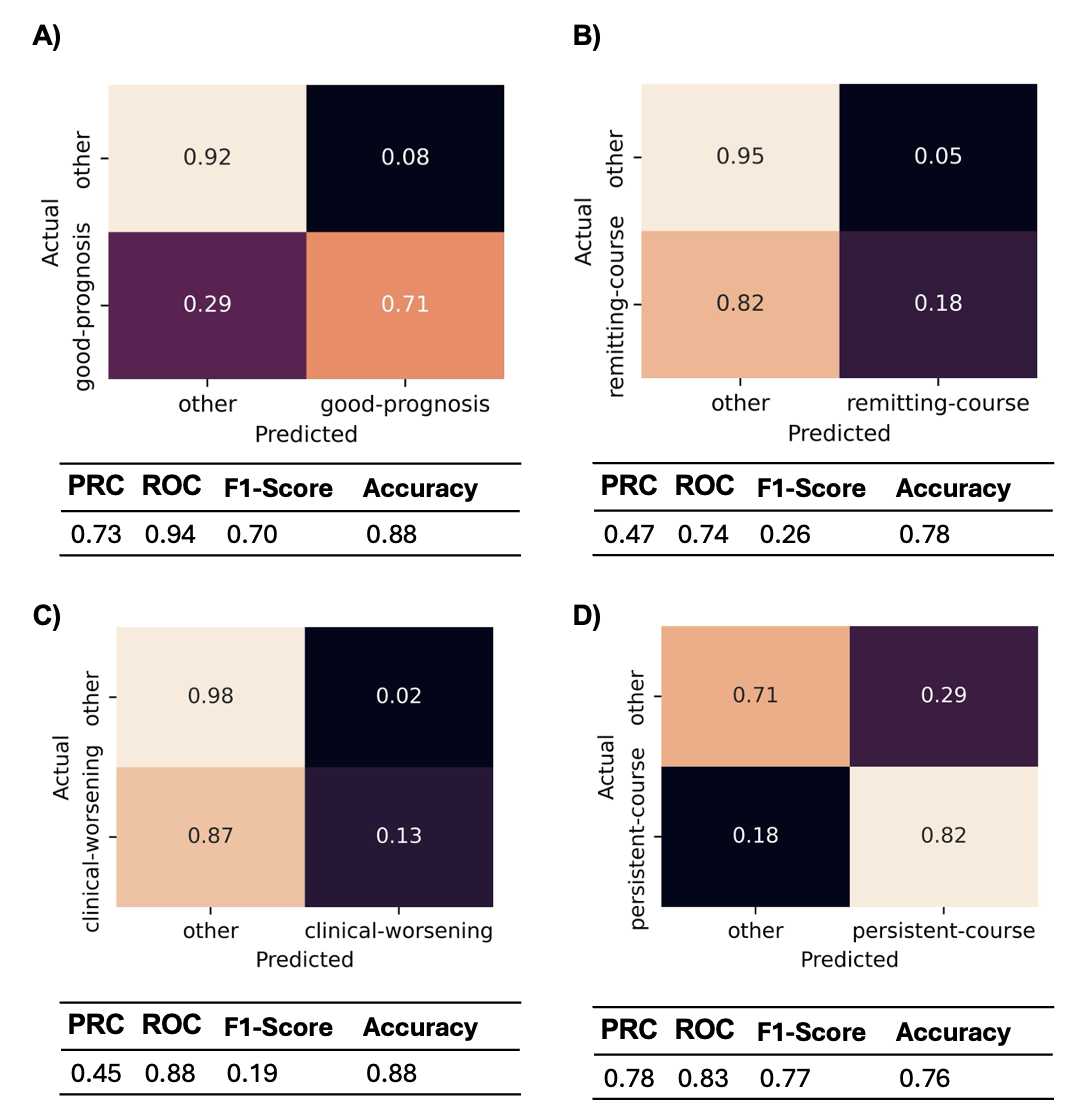


**Figure S3. Confusion Matrices and Performance Metrics of One-vs-Rest Binary Classifiers with default hyperparameters. (A)** Good prognosis, **(B)** Remitting course, **(C)** Clinical worsening, and **(D)** Persistent course labels.


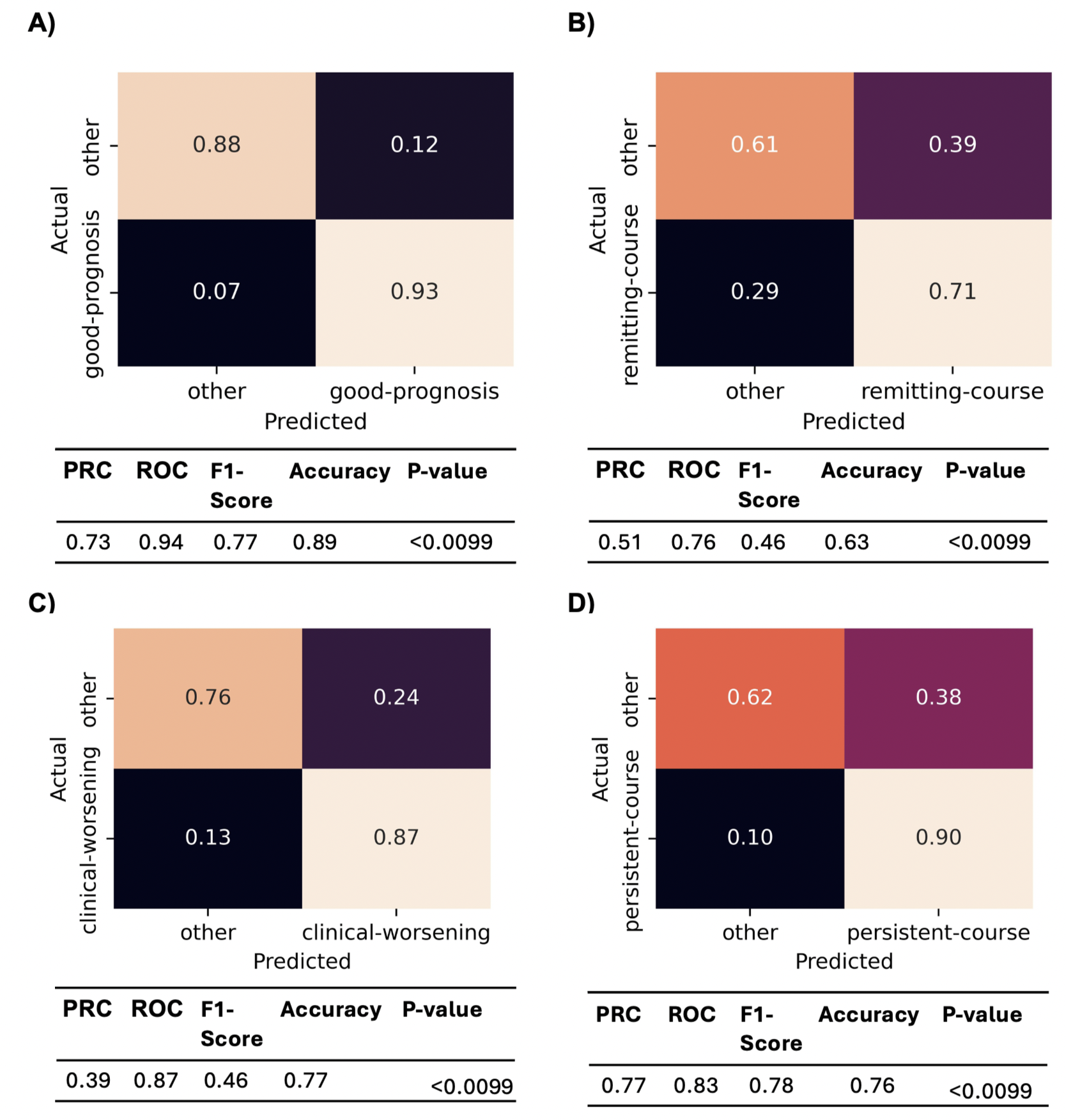


**Figure S4. Confusion Matrices and Performance Metrics of One-vs-Rest Binary Classifiers Optimized for F1.** ‘Good prognosis’ **(A)**, ‘remitting course’ **(B)**, ‘clinical worsening’ **(C)**, and ‘persistent course’ **(D)** labels.

**Supplemental Tables**

**Table S1:** Participant’s Demographics and Clinical Score Stratified by Trajectory

|  | **Good prognosis**  (n = 155) | **Remitting course**  (n = 174) | **Clinical worsening**  (n = 85) | **Persistent course**  (n = 369) |
| --- | --- | --- | --- | --- |
| **Age** (years) | 57.61 (6.23)  [46 – 75] | 57.57 (7.79)  [46 – 77] | 55.49 (6.70)  [46 – 65] | 57.33 (7.26)  [46 – 82] |
| **Monthly income** ($) | 17,335.48 (11,493.94)  [0 - 85000.00] | 13,114.94 (9,200.94)  [0 - 80000.00] | 15,464.71 (11,137.08)  [0 - 90000.00] | 14,164.77 (10,965.29)  [0 - 80000.00] |
| **LEC** | 3.45 (2.16)  [1 - 14] | 10.55 (2.07)  [2 - 15] | 3.87 (2.38)  [1 - 15] | 10.60 (2.14)  [2 - 15] |
| **MSPSS** |  |  |  |  |
| Significant other      Family      Friends | 52.99 (8.75)  [4 - 28]  19.82 (6.26)  [4 - 28]  19.55 (6.56)  [4 - 28] | 36.34 (9.06)  [4 - 24]  10.14 (7.21)  [4 - 24]  7.28 (5.76)  [4 - 24] | 52.48 (6.57)  [4 - 28]  19.13 (7.11)  [4 - 28]  18.72 (7.26)  [4 - 28] | 35.46 (7.52)  [4 - 28]  8.49 (6.69)  [4 - 28]  7.02 (5.54)  [4 - 28] |
| **EQVAS** | 52.99 (8.75)  [15 - 78] | 36.34 (9.07)  [20 - 70] | 52.48 (6.57)  [30 - 70] | 35.46 (7.52)  [20 - 76] |
| **BDQ** | 5.51 (1.98)  [1 - 12] | 10.66 (2.71)  [3 - 12] | 6.47 (2.38)  [5 - 20] | 10.49 (2.50)  [5 - 20] |
| **Gender**  Female  Male | 50 (32.26%)  105 (67.74%) | 62 (35.63%)  112 (64.37%) | 21 (24.71%)  64 (75.29%) | 115 (31.16%)  254 (68.84%) |
| **Education level**  None  Primary  Metric  Graduate | 49 (31.61%)  52 (33.55%)  39 (25.16%)  15 (9.68%) | 75 (43.10%)  59 (33.91%)  28 (16.09%)  12 (6.90%) | 25 (29.41%)  39 (45.88%)  19 (22.35%)  2 (2.35%) | 130 (35.24%)  145 (39.35%)  68 (18.43%)  26 (7.04%) |
| **Marital status**  Married  Single  Separated  Widow | 142 (91.61%)  6 (3.87%)  2 (1.29%)  5 (3.23%) | 162 (93.10%)  4 (2.30%)  1 (0.57%)  7 (4.02%) | 77 (90.59%)  5 (5.88%)  2 (2.35%)  1 (1.18%) | 329 (89.18%)  14 (3.79%)  8 (2.17%)  18 (4.88%) |
| **Employment**  Unemployed  Employed  Retired  Housewife | 9 (5.81%)  79 (50.97%)  19 (12.26%)  48 (31.03%) | 12 (6.90%)  77 (44.25%)  28 (16.09%)  57 (32.76%) | 7 (8.24%)  49 (57.65%)  9 (10.59%)  20 (23.53%) | 39 (10.57%)  192 (52.03%)  37 (10.03%)  101 (27.37%) |
| **History of depression**  No  Yes | 152 (98.06%)  3 (1.94%) | 150 (86.21%)  24 (13.79%) | 84 (98.82%)  1 (1.18%) | 311 (84.32%)  58 (15.72%) |
| **LVF**  No  Yes | 10 (6.45%)  145 (93.55%) | 14 (8.05%)  160 (91.95%) | 5 (5.88%)  80 (94.12%) | 41 (11.11%)  328 (88.86%) |
| **Ischemic cause CHF**  No  Yes | 8 (5.16%)  147 (94.84%) | 7 (4.02%)  167 (95.98%) | 3 (3.53%)  82 (96.47%) | 23 (6.23%)  346 (93.77%) |
| **Diabetes**  No  Yes | 87 (56.13%)  68 (43.87%) | 70 (40.23%)  104 (59.77%) | 41 (48.24%)  44 (51.76%) | 148 (40.11%)  221 (59.89%) |
| **COPD**  No  Yes | 152 (98.06%)  3 (1.94%) | 164 (94.25%)  10 (5.75%) | 84 (98.82%)  1 (1.18%) | 353 (95.67%)  16 (4.34%) |
| **Stroke**  No  Yes | 153 (98.71%)  2 (1.29%) | 165 (94.83%)  9 (5.17%) | 84 (98.82%)  1 (1.18%) | 352 (95.39%)  17 (4.61%) |
| **Renal disease**  No  Yes | 151 (97.42%)  4 (2.58%) | 158 (90.80%)  16 (9.20%) | 81 (95.29%)  4 (4.71%) | 335 (90.76%)  34 (9.21%) |
| **Smoking**  No  Yes | 114 (73.55%)  41 (26.45%) | 118 (67.82%)  56 (32.18%) | 63 (74.12%)  22 (25.88%) | 237 (64.25%)  132 (35.74%) |
| **Prior bypass surgery**  No  Yes | 145 (93.55%)  10 (6.45%) | 163 (93.68%)  11 (6.32%) | 79 (92.94%)  6 (7.06%) | 339 (91.89%)  30 (8.13%) |
| **Prior infarction**  No  Yes | 36 (23.23%)  119 (76.77%) | 22 (12.64%)  152 (87.36%) | 17 (20.00%)  68 (80.00%) | 74 (20.05%)  295 (79.95%) |
| **Ejection Fraction**  I  II  III | 41 (26.45%)  100 (64.52%)  14 (9.03%) | 49 (28.16%)  112 (64.37%)  13 (7.47%) | 26 (30.59%)  49 (57.65%)  10 (11.76%) | 91 (24.68%)  254 (68.84%)  24 (6.50%) |
| **NYHA class**  II  III  IV | 150 (96.77%)  5 (3.23%)  0 (0.00%) | 84 (48.28%)  78 (44.83%)  12 (6.90%) | 67 (78.82%)  18 (21.18%)  0 (0.00%) | 77 (20.87%)  267 (72.35%)  25 (6.78%) |

*Note.* Where appropriate variables are displayed as mean, standard deviation (SD), and range [Max –Min] or frequency count (N) and percentage (%). BDQ = Behavioral Development Questionnaire; CHF = Congestive Heart Failure; COPD = Chronic Obstructive Pulmonary Disease; EQVAS= Euro Quality of Life Visual Analogue Scale; LEC = Life Events Checklist; LVF = Left Ventricular Fraction; MSPSS = Multidimensional Scale of Perceived Social Support; NYHA = New York Heart Association Functional Classification.
